# MiR-25 regulates cell proliferation and metastasis in bladder urothelial carcinoma

**DOI:** 10.7150/jca.62743

**Published:** 2021-09-21

**Authors:** Jin-zhuo Ning, Chuan-min Chu, Yang Du, Li Zuo

**Affiliations:** 1 Department of Urology, Renmin Hospital of Wuhan University, Wuhan, Hubei Province, P.R.China.; 2 Department of Urology, The Third Affiliated Hospital of Naval Medical University, Shanghai, P.R.China.; 3 Department of Urology, The Affiliated Changzhou No. 2 People's Hospital of Nanjing Medical University, Changzhou 213000, Jiangsu Province, P.R.China.

**Keywords:** miR-25, PTEN, bladder cancer, tumorigenesis, prolifertation, metastasis

## Abstract

**Background:** Bladder urothelial carcinoma (BC) is a common malignant tumor with a high incidence. This study aims to explore the role of miR-25 in BC tumorigenesis.

**Material and Methods:** The expression of miR-25 and PTEN were detected in clinical BC tissues. BC cell lines T24 and 5637 were used to transfect miR-25 mimics or inhibitors. Luciferase reporter gene detection confirmed the correlation between miR-25 and PTEN. CCK-8 method and flow cytometry were used to detect cell viability and apoptosis. Cell migration and invasion ability were examined by transwell assays. Western blotting detects the protein levels of PTEN, β-catenin, GSK-3β and p-GSK-3β.

**Results:** MiR-25 and PTEN expression are found to be negatively correlated in BC tissues. Further research confirmed that PTEN is a direct target of miR-25. In addition, the overexpression of miR-25 down-regulates the expression of PTEN, induces cell survival and inhibits apoptosis, while the knockout of miR-25 leads to the opposite result. miR-25 also inhibits the phosphorylation of GSK-3β and β-catenin without changing the total level of GSK-3β. *In vivo* experiments confirmed that miR-25 plays an oncogene's role by regulating the PTEN and Wnt/β-catenin signaling pathways.

**Conclusion:** Our research shows that miR-25 has a negative regulatory effect on the expression of PTEN in clinical specimens and *in vitro*. miR-25 can promote the proliferation of BC cells and induce cell invasion. Therefore, miR-25 may be used as a biomarker to predict the progression of BC.

## Introduction

Bladder urothelial carcinoma (BC) is the second most common genitourinary system cancer in the world. It is estimated that there will be 62,100 new cases and 17,980 deaths in 2020 1. Early detection of BC, the 5-year survival rate is about 94% 2. Therefore, identifying patients at an early stage is very important to improve the prognosis. Extensive research attempts to identify biomarkers for early detection, but these analyses are either limited to high false positive rates or low sensitivity 3. These limitations emphasize the need for new biomarkers.

In the past ten years, a large number of studies have demonstrated the powerful tumor-promoting and anti-tumor functions of microRNAs (miRNAs). miRNAs are a type of small non-coding RNAs that involve in a series of biological function 4. MiRNAs are often dysregulated in bladder cancer, and may promote the development, progression and metastasis of bladder cancer 5. More studies have proved that there are numerous miRNAs circulating in cancer patients, and the levels of these miRNAs have changed, which opens up prospects for the application of circulating miRNAs as biomarkers 6, 7. A miRNA group consisting of 3 miRNAs provides a high diagnostic accuracy rate for gastric cancer 8 and a 5-cycle miRNA marker has been applied as a risk factor of prostate cancer 9.

In our current research, we identified that miR-25, as an oncogene, plays a role in BC cells by promoting cell proliferation, migration and invasion. In addition, our study also confirmed for the first time the mechanism of miR-25 targeting PTEN and miR-25/PTEN axis in BC, which may help the formulation of new treatment strategies for BC.

## Materials and methods

### Clinical tissues

This study was conducted under the permission of Institutional Ethics Committee of the Renmin Hospital of Wuhan University and in accordance with the “Ethical Management Guidelines”. 68 bladder cancer tissue specimens and matched normal specimens were collected from 2017-2019 in Renmin Hospital of Wuhan University. The prior written consent is fully informed and signed by all participants. The clinical data is shown in Table 1.

### Cell lines and cell culture

Human bladder cancer cell lines T24 and 5637 were purchased from the Cell Bank of the Chinese Academy of Sciences (Shanghai). SV-HUC-1 cells were grown in F12K medium, T24 cells were grown in McCoy medium, and 5637 and EJ cells were grown in the smallest RPMI-1640 medium (Gibco, Gaithersburg, MD, USA). Incubate at 37 °C under non-ionic conditions (5% CO2, 95% O2).

### Transfection and Plasmid Construction

The cells were seeded at a density of 1.0 × 10^6^ cells/ml. After 6 hours of culture, according to the manufacturer's instructions, the miR-25 mimic, miR-25 inhibitor and its negative control (NC) were transfected with Lipofectamine 2000 (Invitrogen, Carlsbad, CA) (Biofavor Biotech, Wuhan, China). Before reaching the 90% confluence point, the cells were given 24 hours of starvation for further analysis. The qRT-PCR method was used to amplify the wild-type sequence of pten3'untranslated region (3'UTR) containing miR-25 binding site from T24 and 5637 cells. The 3'UTR sequence of the mutant PTEN gene was obtained by overlap extension qRT-PCR method. Subsequently, all sequences were inserted into the psiCHECK-2 vector (Promega, Madison, WI, USA).

### Luciferase Reporter Assays

In the luciferase reporter gene detection, T24 and 5637 cells were co-transfected with miR-25 mimic and PTEN-3'UTR-luciferase plasmid. After culturing for 48 hours, the cells were collected and lysed. The luciferase activity was measured by a dual luciferase reporter analysis system (Promega, Madison, WI, USA). Each experiment is in triplicate.

### Western blotting

The cells were collected and lysed with RIPA buffer. Protein concentration was measured by the double creatine method (BCA). In short, the equivalent weight of the protein sample (40μg) was separated on 10% SDS-PAGE and then transferred to a PVDF membrane. Subsequently, all cell membranes were incubated with the following primary antibodies against PTEN, β-catenin, GSK-3β, p-GSK-3β (Abcam, Cambridge, UK) overnight at 4 °C. After incubating with the secondary antibody for 1 hour at room temperature, all bands were measured using an ECL system kit (MultiSciences, Hangzhou, China).

### Quantitative real-time PCR

Trizol reagent (InTrogen, CA, USA) was used to extract total RNA from clinical specimens and BC cells. Then, all RNAs were reverse transcribed into cDNA using a reverse transcription kit (Dalian Kaohsiung Biotechnology). Real-time quantitative PCR was performed by applying the Biosystems SYBR Green Hybrid Kit and ABI 7900 real-time PCR system (Applied Biosystems Life Technology, Foster City, CA, USA). The primer sequences are shown in Table 2. The expression of miR-25 or PTEN mRNA was normalized to snRNA-U6 (miRNAs) and GAPDH (mRNAs), respectively.

### Cell Proliferation Assay

The effect of miR-25 on cell viability was measured by CCK-8 method. Cells are cultured in 96-well plates (2 x 103 cells per well) for 24 hours. Cell use 10 μL per well (Sigma-Aldrich, Shanghai, China) 5 mg/mL CCK-8, 37°C for 4h°C. Then discard the medium and add 1% dimethyl sulfoxide (DMSO) to 150 μL. An ELX-800 spectrometer reader (Bio-Tek Instruments, Winooski, USA) was used to detect the absorbance at 490 nm.

### Cell apoptosis assay

Cells were collected and stained with Annexin V and propidium iodide using the FITC/PI Apoptosis Detection kit (BD Biosciences). The apoptotic rate was analyzed by flow cytometry (BD Biosciences).

### Cell Invasion and Migration Assays

Cell invasion and migration were examined using 8-well Transwell plates (Corning) 8-μm-pore membrane with matrix gel (for invasion test) or without matrix gel (for migration test). T24 and 5637 cells were collected 48h after transfection. In short, cells were seeded in the upper matrix gel-coated chamber, and a medium containing 10% fetal calf serum was placed in the lower chamber. After incubation, cells on the upper lumen membrane were wiped away. Stain with 0.2% crystal violet for 30 minutes, and count 5 predetermined fields under a microscope. For invasion assay, the inserts were coated with Matrigel matrix (BD Science, Sparks, MD, USA) diluted in serum-free medium, then incubated at 37 °C for 2 h, remaining procedures were conducted similar to migration assay. All analyses were performed in triplicate.

### Immunohistochemistry

Immunohistochemical staining was used to detect the expression of PTEN. The tissue was fixed with 4% paraformaldehyde, embedded in paraffin, and sectioned with a 3 μm section. After incubation with primary and secondary antibody, the sections were stained with DAB reagent. Primary antibody: anti-PTEN (ab267787, Abcam, CA), secondary antibody: goat anti-rabbit IgG (Biosci, Wuhan China). All slices were photographed at a magnification of × 400.

### Tumor xenograft formation assay

6-week-old male BALB/C nude mice were from the Animal Center of Wuhan University People's Hospital. Studies were approved by the Animal Care and Use Committee of Wuhan University. T24 cells transfected with miR-25 inhibitor or negative control cells were subcutaneously inoculated into the left abdominal wall of nude mice (n=4/group, 1×106 cells per cell). Check the tumor growth every 5 days, and use the following formula to calculate the tumor volume: Volume = (length × width^2^) / 2(mm^3^). The animals were sacrificed 4 weeks after the inoculation. The xenografts were dissected and analyzed in subsequent studies.

### Statistical Analysis

All data are expressed as mean ± standard deviation. Two-tailed Student's *t*-test and t-test were used to evaluate differences. χ^2^ was tested as appropriate. P value less than or equal to 0.05 is considered statistically significant. Each experiment is in triplicate. Use spss20.0 (SPSS, Chicago, IL) for statistical analysis.

## Results

### miR-25 negatively correlates with the expression of PTEN in BC tissues

**Figure 1:**
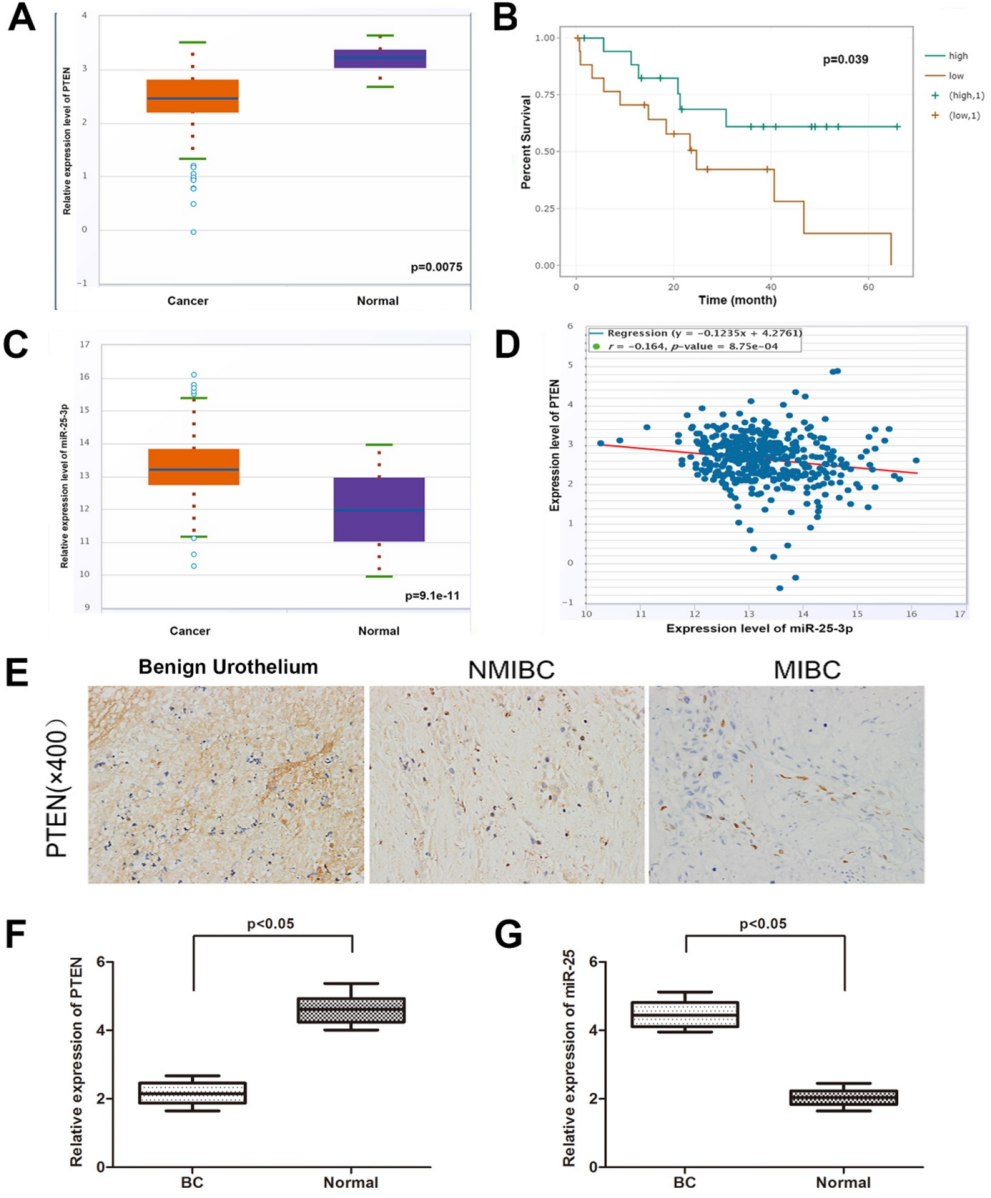
** The correlation between miR-25 and PTEN in BC samples. (A)** TCGA data from Starbase shows the expression of PTEN in BC tissues and paired normal tissues. **(B)** Kaplan-Meyer analysis of PTEN expression and overall survival of BC patients. The correlation of rate **(C)** data from Starbase 2.0 shows the expression of miR-25 in BC tissues and paired normal tissues. **(D)** Data from Starbase 2.0 shows that the expression of miR-25 is negatively correlated with PTEN expression. **(E)** Immunohistochemical staining of PTEN in normal bladder tissue, NMIBC tissue and MIBC tissue. **(F)** Box diagram shows the relative expression of PTEN in BC tissues and paired normal tissues. **(G)** Box diagram shows miR-25 in BC tissues and paired normal tissues Relative expression in.

In order to determine the expression of PTEN in BC and normal tissues, we explored the expression of PTEN in the TCGA data portal of starbasever2.0. As shown in Figure 1A, PTEN was significantly reduced in BC tissues compared to normal tissues. In addition, data from Starbase 2.0 showed that compared with patients with high PTEN expression, patients with low PTEN expression had a worse overall survival time (Figure 1B). Then, we tested the expression of PTEN in NMIBC, MIBC and normal tissues (Figure 1E). The result is consistent with the database result. On the other hand, the database showed that the expression of miR-25 in BC samples was higher than that in matched normal samples (Figure 1C). Then we verified this result in the same way with the organization of our department (Figure 1F, G). Pearson's correlation analysis showed that the expression of miR-25 was negatively correlated with the expression of PTEN (Figure 1D).

### MiR-25 directly targets PTEN and negatively regulates PTEN expression *in vitro*

**Figure 2:**
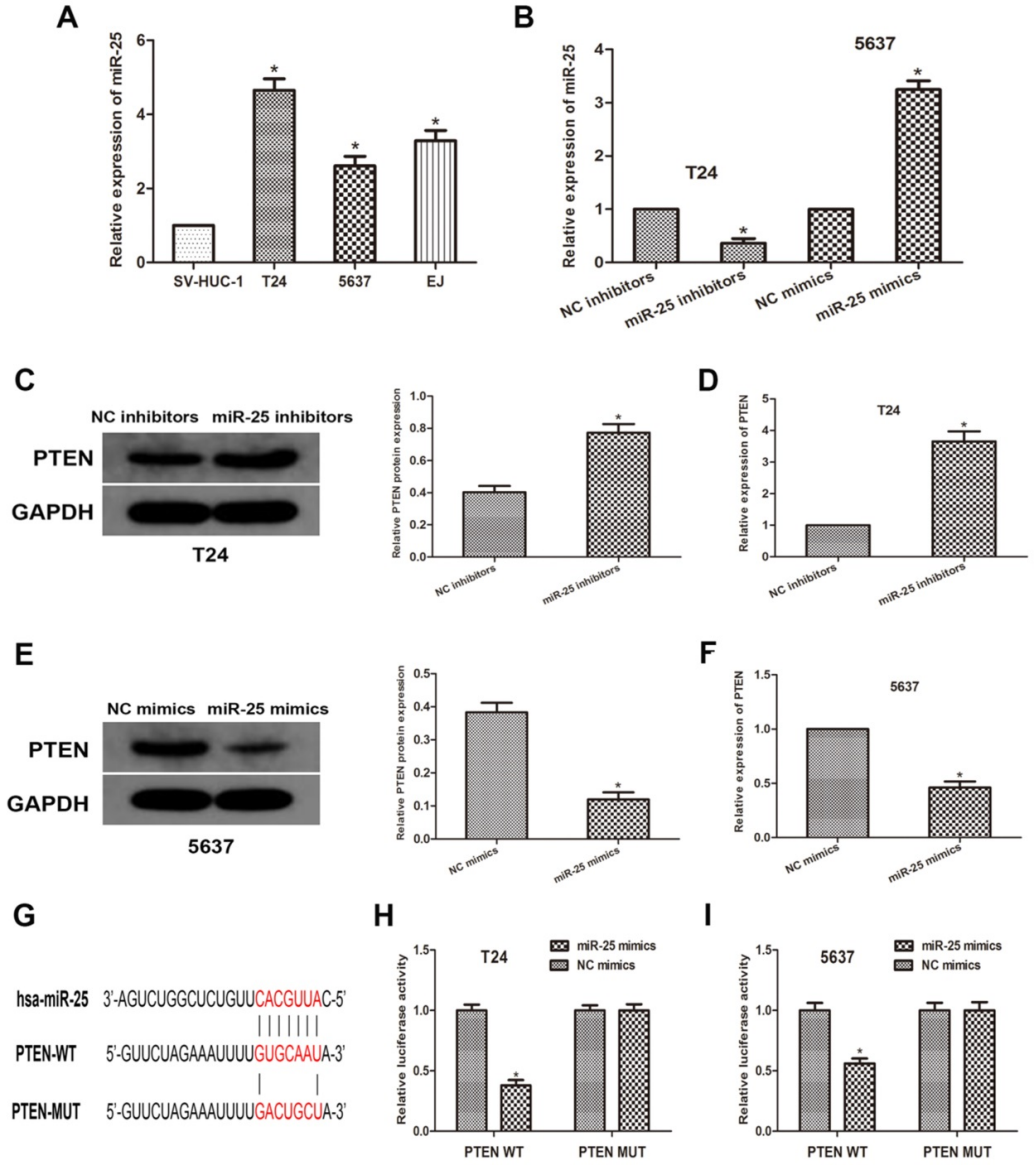
** MiR-25 directly regulates the expression of PTEN *in vitro*. (A)** The expression of miR-25 in bladder cancer cell lines (T24, 5637 and EJ) and human bladder epithelial cells (SV-HUC-1) (*P<0.05) vs. SV-HUC-1 group). **(B)** qRT-PCR detection of miR- in T24 cells transfected with miR-25 inhibitor and 5637 cells transfected with miR-25 mimic Expression of 25 (*P<0.05 compared with the corresponding NC group). **(C-F)** Western blot and qRT of PTEN expression of miR-25 inhibitor transfected in T24 cells and miR-25 mimic transfected in 5637 cells PCR analysis (*Compared with the corresponding NC group, P<0.05). **(G)** Sequence alignment of miR-25 binding site in pten3'UTR and its mutation sequence in luciferase reporter gene detection. **(H, I)** Luciferase reporter analysis was performed in T24 and 5637 cells co-transfected with miR-25 mimic and reporter vector containing PTEN 3'UTR or mutant PTEN 3'UTR. The relative activity of luciferase is introduced (*compared with the corresponding NC simulation group, P<0.05).

To further explore the relationship between miR-25 and PTEN, we used human normal bladder cell lines SV-HUC-1 and BCa cell lines T24, 5637 and EJ. We transfected T24 and 5637 cells with miR-525 mimics or inhibitors, and then obtained miR-25 overexpression or knockdown cells (Figure 2A, B). Through western blots, we measured the expression of PTEN (Figure 2C, D). The data revealed that the expression of PTEN was remarkably reduced in miR-25 overexpressing cells, while the expression in miR-25 knockout cells was moderately increased (Figure 2E, F). Subsequently, we used open access databases (Targetscan, miRanda, and miRwalk2.0) to predict that PTEN is a downstream target of miR-25, and determined a putative binding site for miR-25 in the 3'UTR of PTEN (Figure 2G). To confirm this prediction, a luciferase reporter analysis was performed. The results showed that in the cells overexpressing miR-25, the reporter activity of pten3'UTR was significantly inhibited, while that kept stable in the 3'UTR mutated group (Figure 2H, I). In sum, these findings indicate that miR-25 can negatively regulate the expression of PTEN by directly binding to PTEN.

### miR-25 regulates the progression of BC *in vitro*

**Figure 3:**
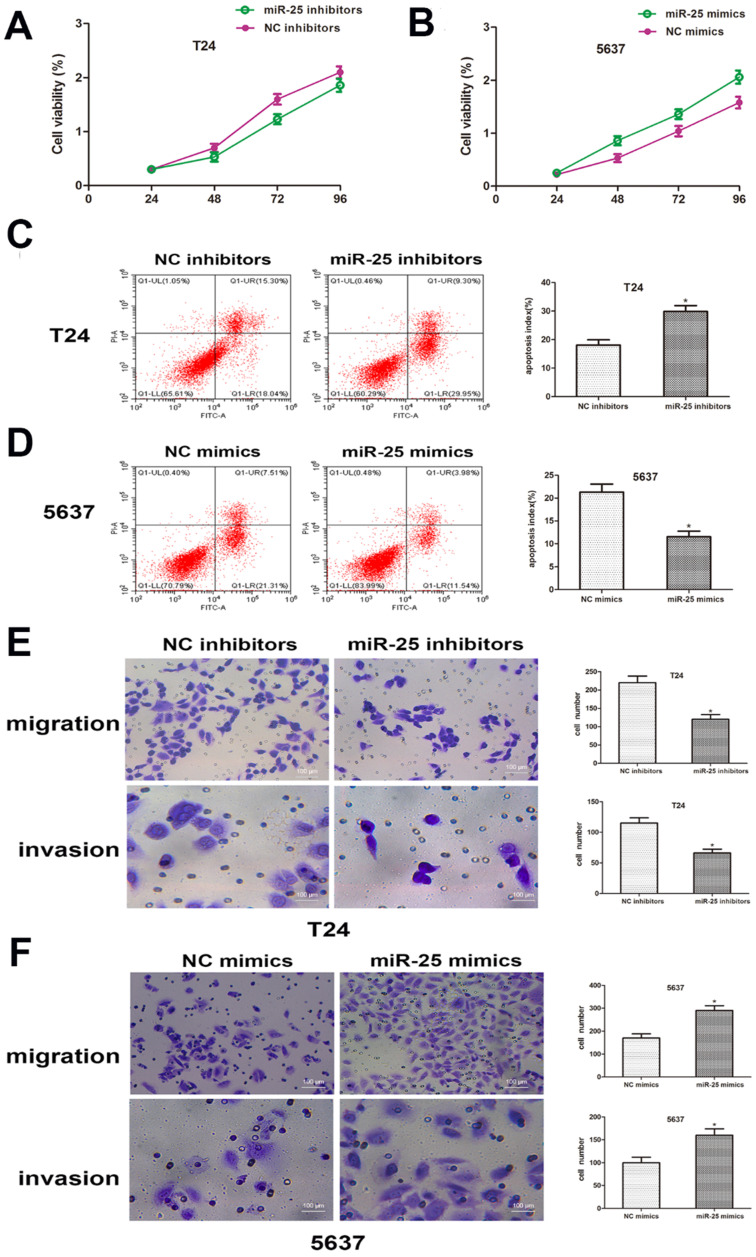
** miR-25 regulates the malignant phenotypes of BC *in vitro.* (A, B)** CCK-8 assay was performed to determine the cell viability of T24 cells transfected with miR-25 inhibitor and 5637 cells transfected with miR-25 mimic. Detect absorbance values at 24, 48, 72, and 96 hours after transfection (*compared with the corresponding NC group, P<0.05). **(C, D)** Flow cytometry to detect miR-25 inhibitor-transfected T24 cells and transfected Apoptosis of 5637 cells infected with miR-25 analogues (*P<0.05 compared with the corresponding NC group). **(E, F)** After transfection of T24 and 5637 cells, cross-well migration and invasion experiments were performed to detect cell Migration and invasion capabilities. The number of cells in 5 random areas was counted in units of 200 × magnification (*P<0.05 compared with the corresponding NC group).

In order to clarify the role of miR-25 in the proliferation of BC cells, CCK-8 analysis was performed. The results showed that compared with the control group, the viability of T24 cells transfected with miR-25 inhibitor was significantly inhibited, while the viability of T24 cells transfected with 5637 cells was significantly inhibited. The miR-590 mimic in in strongly promoted cell viability (Figure 3A, B). In addition, we also studied the role of miR-25 in BC cell apoptosis. Flow cytometry data showed that compared with the control group, the knockout of miR-25 resulted in a significant rate of apoptosis, and this inhibition was eliminated by the use of miR-25 mimics (Figure 3C, D). In addition, we also tested the effect of miR-25 on cell migration and invasion. The results of transwell experiment showed that the number of miR-25 high expression group was significantly higher than that of NC group, while the number of miR-25 knockout group was significantly reduced (Figure 3E, F). In conclusion, our research proves that miR-25 is an oncogene in BC cells.

### miR-25 involves in the regulation of Wnt/β-catenin signaling pathway

**Figure 4:**
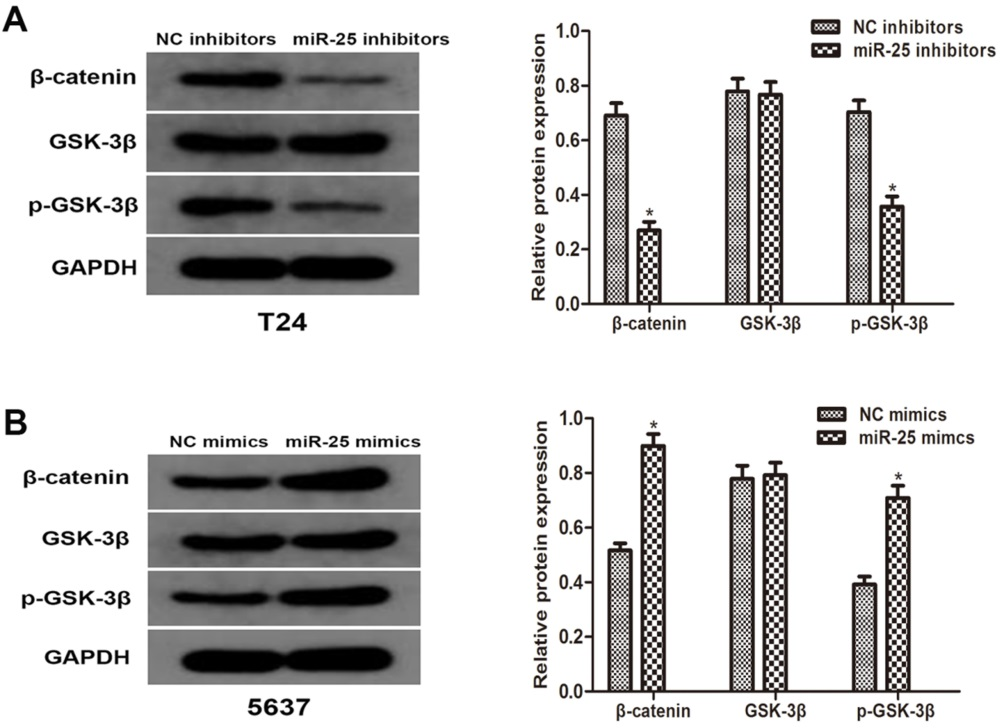
** miR-25 regulates Wnt/β-catenin signaling pathway *in vitro.* (A, B)** The protein levels of β-catenin, p-GSK-3β and GSK-3β in T24 cells transfected with miR-25 inhibitors and 5637 cells transfected with miR-25 mimics were detected by western blot. GAPDH was used as an internal control (*P<0.05 vs. respective NC group).

To better understand the molecular mechanism of miR-25 in BC, we analyzed the phosphorylation level of GSK-3β and β-catenin. Western blotting results showed that the overexpression of miR-25 significantly increased the phosphorylation of GSK-3β and β-catenin. The total level of GSK-3β is consistent with the expression of miR-25. These results indicate that miR-25 is involved in the regulation of Wnt/β-catenin signaling pathway and may be an important factor in the occurrence of tumors in BC cells (Figure 4A, B).

### MiR-25 regulates tumor growth of BC *in vivo*

**Figure 5:**
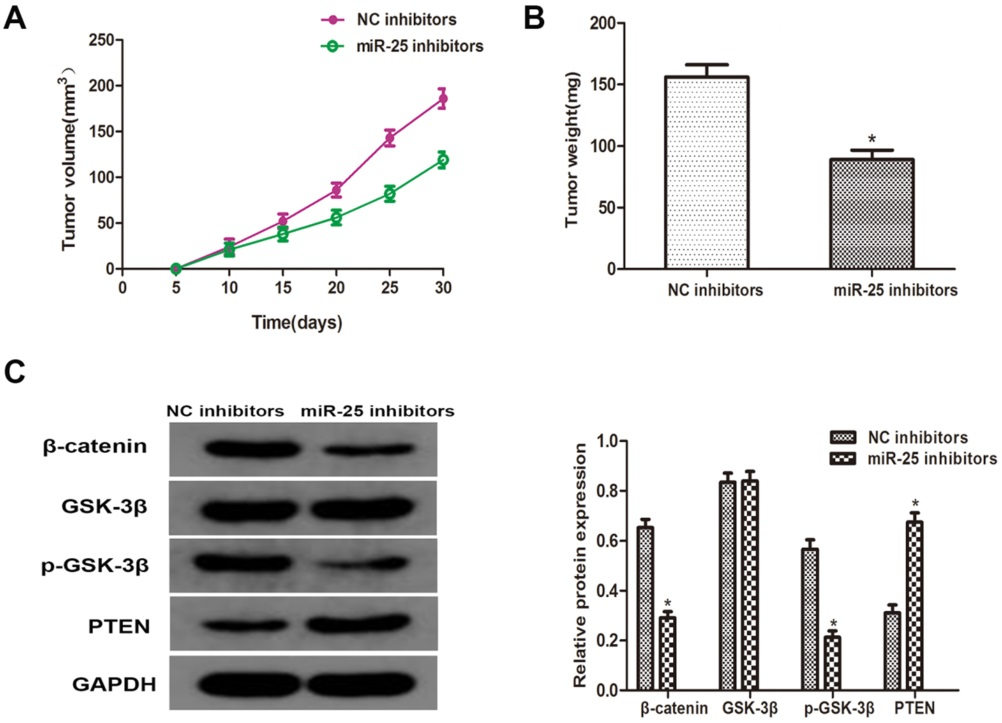
**MiR-25 regulates cell prolifertaion of BC *in vivo*. (A)** Calculate tumor volume every 5 days from day 5 to day 28 after inoculation (*Compared with NC inhibitor group P<0.05). **(B)** Measure tumor weight of xenograft (*compared with NC inhibitor group Ratio P<0.05). **(C)** The protein level of PTEN, β-catenin, p-GSK-3β GSK-3β westernblot to detect the cells transfected with miR-25 inhibitor. GAPDH was used as an internal control (*P<0.05 compared with the corresponding NC inhibitor group).

To verify whether miR-25 can induce the occurrence and development of BC *in vivo*, T24 cells transfected with miR-25 inhibitor were subcutaneously inoculated into BALB/c nude mice. Four weeks after the inoculation, the xenograft tumor was removed and left for further analysis. The tumor volume and weight were measured, and the results showed that compared with the NC group, miR-25 knockout significantly inhibited tumor growth (Figure 5A, B). Subsequently, the protein levels of PTEN, β-catenin, p-GSK-3β and GSK-3β were detected. The results showed that the expression of PTEN in the miR-25 knockdown group was significantly higher than that in the NC group, while the expression of p-GSK-3 was significantly higher than that in the NC group, and β-catenin was significantly inhibited (Figure 5C). The above results suggest that miR-25 may be an oncogene in the occurrence and development of BC.

## Discussion

It is well known that miRNA regulates gene expression at the post-transcriptional level through translational inhibition and mRNA instability. More and more studies have shown that miRNAs act as oncogenes or tumor suppressor genes in different cancers 10, 11. According to reports, miR-25 promotes the proliferation of triple-negative breast cancer by targeting BTG2 12. It has also been reported that miR-25 can play a tumor suppressor role in highly metastatic PCSCs through direct functional interaction with the 3'-untranslated region 13. The latest research shows that miR-25 can be used as a biomarker for the prognosis of hepatocellular cancer 14. However, the role of miR-25 in BC and its molecular mechanism are still unclear. In this study, we found that miR-25 and PTEN are negatively correlated in BC tissues. Compared with matched normal tissues, MiR-25 was significantly up-regulated in BC tissues, while PTEN was significantly reduced in tumors. Subsequently, we verified the binding relationship between miR-25 and PTEN through an open-access bioinformatics database, and determined that PTEN is a direct downstream target of miR-25 through luciferase reporter analysis. In addition, our research shows that the overexpression of miR-25 significantly promotes the proliferation, migration and invasion of cells, while the knockout of miR-25 eliminates the above effects. Our findings are consistent with previous reports, suggesting that miR-25 acts as an oncogene in BC cells, and may be used as a biomarker for the diagnosis and prognosis of BC.

Wnt/β-Catenin signaling pathway has been extensively studied in various cancers. According to reports, the Wnt/β-catenin pathway is regulated by many factors in bladder cancer cells, including microRNAs. Therefore, it is important to determine the upstream regulator of Wnt/β-catenin signal transduction in the treatment of breast cancer. In our current research, we use bioinformatics methods to predict that miR-25 will directly bind to PTEN, and then use luciferase reporter gene analysis to verify this correlation. Subsequently, we studied the effect of miR-25 on the expression of PTEN and Wnt/β-catenin signal. Our results show that the overexpression of miR-25 significantly inhibits the expression of PTEN and triggers the activation of β-catenin and phosphorylation of GSK-3β. On the other hand, miR-25 gene knockout significantly induces the up-regulation of PTEN expression, suggesting miR-25 plays an oncogene role in the occurrence and development of BC.

## Conclusion

The current study revealed that miR-25 may regulate the progression of BC cells by promoting cell proliferative and invasive capacity, and this regulatory effect may be ascribed to the regulation of PTEN and Wnt/β-catenin signaling pathway. Our findings suggested that miR-25 has a potential to be used as a diagnostic biomarker in the progression of BC.

## Figures and Tables

**Table 1:** Relative miR-25 expression and the clinical characteristics of 68 patients with BC

Variable	Group	MiR-25 expression			P value
		Low	High	Total	
Sex	Male	35	6	41	0.983
	Female	23	4	27	
Age	<70	15	21	36	0.582
	≥60	13	19	32	
Tumor grade	NMIBC	13	27	40	<0.001
	MIBC	8	20	28	
Tumor size	<3 cm	13	24	37	<0.001
	≥3 cm	9	22	31	

**Table 2:** RT-PCR primer sequences

GENE	Primer sequences (5'-3')
PTEN	F: TCTAGTGCATCGCTCCTCGT
	R: TTACGAACGTTACCTCAGG
miR-25	F: GGCTTACACGCGACAGCTAGC
	R: CCGATTTGCGGAGGTGGAGCT
U6 snRNA	F: ACGGCGTCGGTTCGACATAGCA
	R: TACGGCCATCCGTAAGCGTGTG
GAPDH	F: TGCCTGATTGCGAGTTCACGGCATG
	R: GCTGCATGGCCATTAGCGCTCCACC
